# Interrater reliability of the Fugl-Meyer Motor assessment in stroke patients: a quality management project within the ESTREL study

**DOI:** 10.3389/fneur.2024.1335375

**Published:** 2024-04-08

**Authors:** Karin Wiesner, Anne Schwarz, Louisa Meya, Josefin Emelie Kaufmann, Christopher Traenka, Andreas Rüdiger Luft, Jeremia Philipp Oskar Held, Stefan Engelter

**Affiliations:** ^1^Neurorehabilitation and Neurology, University Department of Geriatric Medicine FELIX PLATTER, University of Basel, Basel, Switzerland; ^2^Department of Health Professions, Bern University of Applied Sciences, Bern, Switzerland; ^3^Division of Vascular Neurology and Neurorehabilitation, Department of Neurology, University of Zurich and University Hospital Zurich, Zurich, Switzerland; ^4^Department of Neurology and Department of Clinical Research, University of Basel and University Hospital Basel, Basel, Switzerland; ^5^Cereneo Center of Neurology and Rehabilitation, Zurich, Switzerland; ^6^Valens Clinics, Reha Center Triemli, Vitznau, Switzerland

**Keywords:** Fugl-Meyer assessment, stroke, motor disorders, recovery, rehabilitation, reliability, training

## Abstract

**Introduction:**

The Fugl-Meyer Motor Assessment (FMMA) is recommended for evaluating stroke motor recovery in clinical practice and research. However, its widespread use requires refined reliability data, particularly across different health professions. We therefore investigated the interrater reliability of the FMMA scored by a physical therapist and a physician using video recordings of stroke patients.

**Methods:**

The FMMA videos of 50 individuals 3 months post stroke (28 females, mean age 71.64 years, median National Institutes of Health Stroke Scale score 3.00) participating in the ESTREL trial (Enhancement of Stroke Rehabilitation with Levodopa: a randomized placebo-controlled trial) were independently scored by two experienced assessors (i.e., a physical therapist and a physician) with specific training to ensure consistency. As primary endpoint, the interrater reliability was calculated for the total scores of the entire FMMA and the total scores of the FMMA for the upper and lower extremities using intraclass correlation coefficients (ICC). In addition, Spearman’s rank order correlation coefficients (Spearman’s rho) were calculated for the total score and subscale levels. Secondary endpoints included the FMMA item scores using percentage agreement, weighted Cohen’s kappa coefficients, and Gwet’s AC1/AC2 coefficients.

**Results:**

ICCs were 0.98 (95% confidence intervals (CI) 0.96–0.99) for the total scores of the entire FMMA, 0.98 (95% CI 0.96–0.99) for the total scores of the FMMA for the upper extremity, and 0.85 (95% CI 0.70–0.92) for the total scores of the FMMA for the lower extremity. Spearman’s rho ranged from 0.61 to 0.94 for total and subscale scores. The interrater reliability at the item level of the FMMA showed (i) percentage agreement values with a median of 77% (range 44–100%), (ii) weighted Cohen’s kappa coefficients with a median of 0.69 (range 0.00–0.98) and (iii) Gwet’s AC1/AC2 coefficients with a median of 0.84 (range 0.42–0.98).

**Discussion and conclusion:**

The FMMA appears to be a highly reliable measuring instrument at the overall score level for assessors from different health professions. The FMMA total scores seem to be suitable for the quantitative measurement of stroke recovery in both clinical practice and research, although there is potential for improvement at the item level.

## Introduction

Motor impairment is one of the most important disabilities associated with stroke and can significantly affect the quality of life (1). Muscle weakness, abnormal synergy, and spasticity are among the motor deficits commonly assessed in stroke patients (2). Considering the repair processes, measuring motor recovery after stroke is very important. The Fugl-Meyer Motor Assessment (FMMA) (3) is strongly recommended as a clinical and research tool for the evaluation of changes in motor impairment after stroke (4). It was a key component of the assessment recommendations for improving the methodology of adult rehabilitation and recovery trials (5) and clinical motor rehabilitation (6), which should be repeated at different measurement time points. The inclusion of the upper extremity FMMA (FMMA-UE) in further recommendations for outcome measurement after stroke has confirmed its importance (7, 8).

The maximum total score per side is 66 points for the FMMA-UE and 34 points for the lower extremity FMMA (FMMA-LE) (4). The FMMA items are rated on an ordinal scale with the scores 0 = cannot perform, 1 = performs partially and 2 = performs fully (4). The practical implementation of the test and the assessment of its individual items require standardized, sound training as well as routine. These aspects can be promoted by a uniform test version in the different languages of the respective countries of application. Upon completion of the present project, standardized FMMA test forms translated into more than 10 different languages were available [e.g., at https://www.gu.se/en/neuroscience-physiology/fugl-meyer-assessment (9)]. However, to the best of our knowledge, no standardized, validated German version of the test is currently available. Therefore, we developed an adapted German version of the assessment, based on the original article and protocols of the University of Gothenburg (3, 10, 11). The corresponding assessment forms can be found in the Supplementary Table S1. The interprofessional application of this German version of the FMMA into clinical trials requires good psychometric properties in terms of the validation process.

A high interrater reliability of the German version of the FMMA across different health professions is essential for the use of the assessment in clinical studies, but also for its application in daily rehabilitation practice. The English version of the FMMA showed excellent intra- and interrater reliability (4). Platz et al. (12) found a very high interrater reliability of the FMMA-UE with intraclass correlation coefficients (ICC) based on video recordings. In the Sullivan et al. (13) study, interrater agreement between expert and therapist raters using video recordings was high for the FMMA total scores with an ICC value of 0.98 as well as for total scores of the FMMA-UE with 0.99 and moderate to high for the FMMA-LE total scores with 0.91. Based in part on the strong evidence for validity, reliability, responsiveness, and clinical utility, the FMMA-UE was incorporated into the core set of European evidence-based recommendations for Clinical Assessment of Upper Limb In Neurorehabilitation (CAULIN) (7).

In this context, refined reliability data and the availability of transculturally adapted, validated FMMA versions in different languages are even more important. Investigating the interrater reliability of new FMMA versions using sufficiently large samples is a relevant component in this regard. Therefore, we aimed to investigate (i) the interrater reliability of the German FMMA across health professions and (ii) the comparability of the psychometric properties of the German FMMA with those of the English version.

## Materials and methods

### Project objectives and design

The aim of this research project was to study the interrater reliability of the German version of the FMMA in terms of its consistent and accurate application across health professions. The FMMA is used in the ongoing Swiss multicentre ESTREL trial (Enhancement of Stroke Rehabilitation with Levodopa: a randomized placebo-controlled trial, BASEC-number 2018–02021, ClinicalTrials.gov NCT03735901) (14), in which the current reliability study with a cross-sectional design was embedded.

### Study population and procedure

All patients in this study had a video recorded FMMA at their regular three-month visit as part of their participation in ESTREL (14, 15). In brief, ESTREL investigates whether Levodopa, compared to placebo, given in addition to standardized rehabilitation based on the principles of motor learning, is associated with a patient-relevant enhancement of functional recovery in acute ischemic or haemorrhagic stroke patients, as measured by the FMMA after 3 months (14, 15).

The present project followed the Guidelines for Reporting Reliability and Agreement Studies (GRRAS) (16). As a preparatory step, an extensive literature research on relevant FMMA publications in the English-speaking world was conducted. After the selection of adequate reference literature, different FMMA versions were analyzed in detail and their contents were precisely compared. Between December 2019 and May 2021, FMMA video recordings of the three-month visit of ESTREL stroke patients were performed at the two best recruiting centres, Basel and Zurich, Switzerland. The FMMA was applied in an outpatient visit setting, in most cases by the first author (KW).

**Figure 1:**
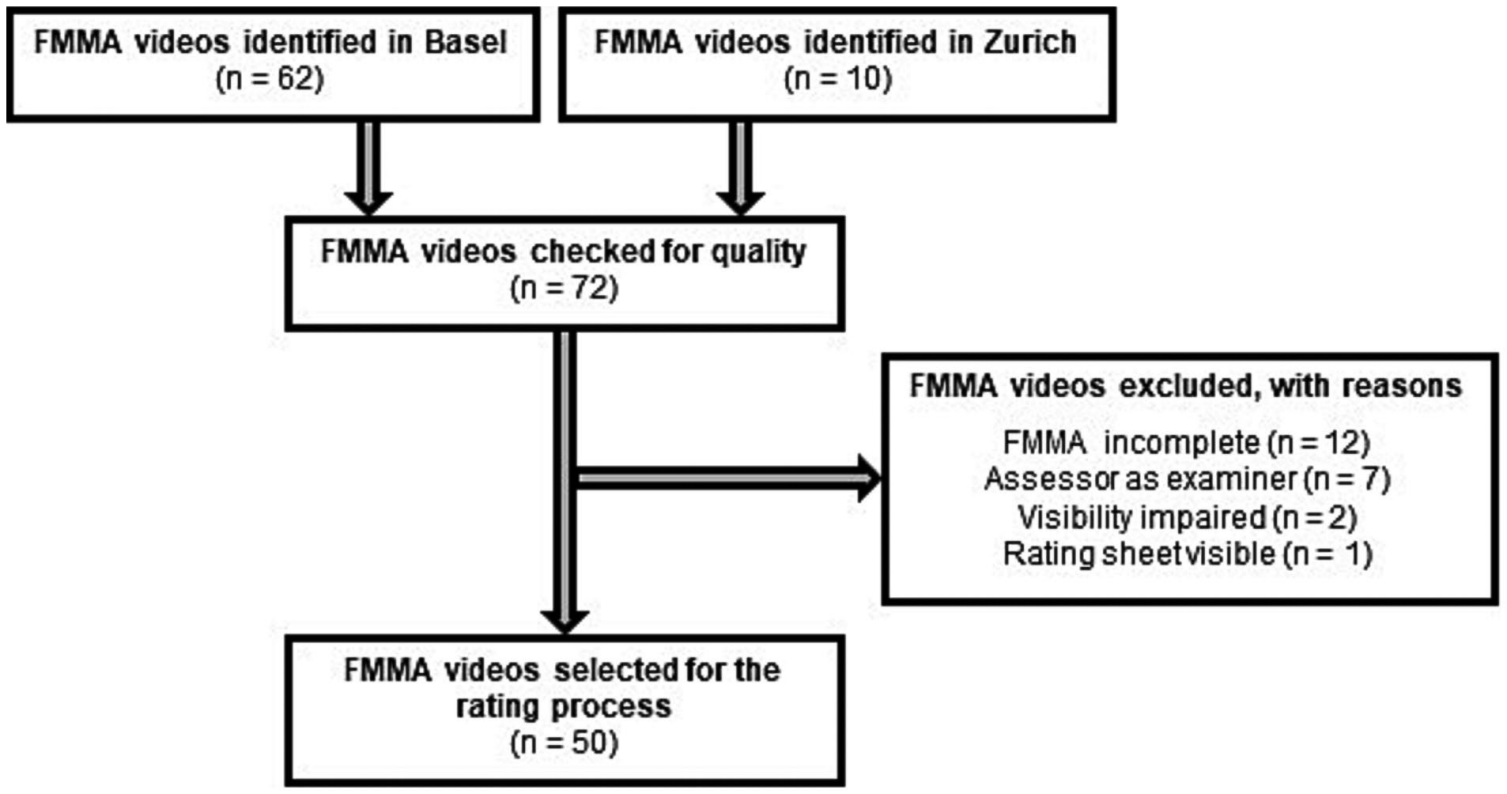
Flow chart of the selection process for FMMA video recordings. FMMA, Fugl-Meyer Motor Assessment; n, number of subjects.

The sampling method of the recordings was consecutive, following a standardized procedure. Eligibility criteria: We took the first 72 available FMMA videos from ESTREL participants who were eligible for the trial (14). Of these, 62 videos were identified in Basel and 10 in Zurich. The first author (KW) performed a quality check of all collected videos based on the criteria of (i) completeness, (ii) visibility of the entire examination, and (iii) potential source of bias. Video recordings were excluded, if (a) the FMMA was incomplete, (b) a FMMA subscale was not fully visible, and (c) the evaluation sheet with the FMMA ratings was visible on the video. In addition, recordings were excluded if one of the assessors of the videos was the FMMA examiner being videotaped. A flowchart of the video selection process is presented in Figure 1.

### Independent assessors

**Figure 2:**
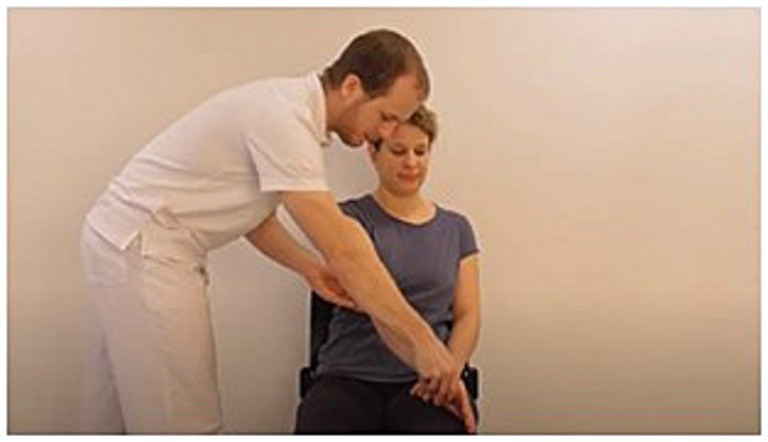
Example illustration from FMMA training for assessors: FMMA-UE. FMMA-UE, Fugl-Meyer motor assessment upper extremity.

Two independent assessors – one from each participating centre – rated the FMMA videos. Rating was limited to the hemiparetic side in each case. The assessors consisted of one research physician (LM) and one research physiotherapist (AS) from the two different centres, each with a master’s degree and clinical experience – who met the following criteria: First, both assessors had participated at least twice in a standardized, four-hour in-person FMMA training course by an FMMA expert (JH), based on the German version of the FMMA (see Figure 2, FMMA training). Second, both assessors had applied the German version at least 50 times on stroke patients in a standardized setting.

**Figure 3:**
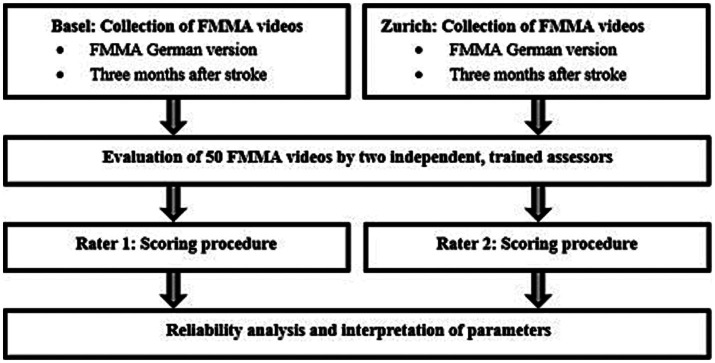
Flow chart of the study procedure. FMMA, Fugl-Meyer Motor assessment.

The assessors scored the videos separately in space and time, and independently of each other and other study personnel. The scores were directly entered in coded electronic case report forms (eCRF) of the German version of the FMMA within the secure web application REDCap (Research Electronic Data Capture) (17). Regarding clinical information, the assessors were unaware of the initial stroke severity, including the FMMA scores at baseline, but were not blinded to the medical history of the subjects in the video recordings. Both assessors had the same access to on-site training and additional video tutorials for recapitulation. They were given additional guidance and explanation on how to proceed in special situations where FMMA items could not be completed for non-stroke-related reasons (e.g., due to pain) or where items were incomplete on video. The flow chart of the study procedure can be found in Figure 3.

### Data recording and confidentiality

The videos were recorded with a GoPro camera, GoPro Incorporation (San Mateo, California, US). Camera positions (heights, distances) were exactly defined in written form for the FMMA-UE and the FMMA-LE and are presented in the Supplementary Figure S1. The storage of all health-related personal data was protected by appropriate operational and organizational measures in accordance with Article 18 of the Ordinance on Clinical Trials in Human Research of the Swiss Confederation (18).

The informed consent form for the ESTREL trial specifies that the FMMA tests may only be recorded and used for internal research purposes in order to conduct a thorough evaluation.

### Statistical reliability analysis

A sample size of 50 subjects was recommended for reliability studies in order to reasonably determine kappa values (19). In our project, we followed this recommendation, as well as appropriate reference studies that included between 10 and 60 individuals after stroke in their reliability analyses (12, 13, 20–26).

Our primary endpoint was the interrater reliability of the FMMA, calculated for the total scores of the entire FMMA and the total scores of the FMMA for the affected extremities using ICCs with the corresponding 95% confidence intervals (CI). The following ICC form fitted the model best: Two-way mixed effects, absolute agreement, multiple raters/measurements (27, 28). ICC values were also calculated for the FMMA subscales, as these parameters were recommended for use with continuous variables (19). For comparison with the reference literature, ICCs were calculated for all FMMA subscales. Since it is questionable whether the ICC – as a parameter for continuous variables (19) – is suitable for variables with few levels, the ICC was not considered as the only parameter for the coordination subscales (three items/0–6 levels) of the FMMA and for the wrist (five items/0–10 levels) and hand (seven items/0–14) subscales of the FMMA-UE. For these subscales, weighted Cohen’s kappa values with associated CIs were calculated. Since the data were non-parametric, Spearman’s rank-order correlation coefficients (Spearman’s rho values) with appropriate *p*-values and 95% CI were calculated to document the strength of association for total and subscale evaluations between assessor 1 and assessor 2.

Several statistical procedures formed the secondary endpoints for assessing the reliability of the FMMA at the item level: (i) Percentage agreement values between the two ratings were calculated for all 50 FMMA individual tasks of the affected extremities. (ii) Weighted Cohen’s kappa (29, 30) values and corresponding 95% CI were obtained from the FMMA ordinal variables. (iii) Gwet’s AC1/AC2 coefficients with corresponding 95% CIs were calculated at the item level in addition to the weighted Cohen’s kappa values.

Statistical procedures to determine all end points were performed using RStudio software, version 1.2.1335.

### Evaluation of parameters

Reliability parameters were categorized according to appropriate classifications (see Supplementary Tables S2, S3): We applied the 95% CIs of the ICC estimates for interpretation instead of the ICC estimates themselves (27) and used the Landis and Koch (31) classification for the weighted Cohen’s kappa and the Gwet’s AC1/AC2 values to compare the results of the German FMMA with those of previously published studies.

## Results

Fifty video recordings were eligible to study the interrater reliability of the German FMMA version (Figure 1). There were no missing data that affected the statistical analysis.

### Patient characteristics

50 individuals with stroke were recorded 3 months ±14 days after randomization in the ESTREL trial. 28 of the participants were female, the mean age was 71.64 years, and the median National Institutes of Health Stroke Scale (NIHSS) score was 3.00. All patient demographic and clinical characteristics are presented in Table 1.

**Table 1:** Patient demographic and clinical characteristics.

Person and stroke-related characteristics (n = 50)	Value
Age, mean (SD)	71.64 (±11.38)
Gender, males (%)	22 (44)
Handedness, left (%)	3 (6)
Body height, mean (SD)	167.56 (±8.73)
Body weight, mean (SD)	71.36 (±12.20)
Pre-stroke living situation, living at home (%)	50 (100)
Stroke type, haemorrhagic (%)	8 (16)
Recanalisation therapy, yes (%)	17 (34)
Affected body side, left (%)	30 (60)
NIHSS, median (Q1-3)	3.00 (1.00–4.00)
Pre-stroke mRS estimate, median (Q1-3)	0.00 (0.00–0.00)
mRS, median (Q1-3)	3.00 (0.00–3.75)
FAC, median (Q1-3)	4.00 (4.00–5.00)

FAC, functional ambulance categories; mRS, modified Ranking Scale; n, number of subjects; NIHSS, National Institute of Health Stroke Scale; Q, quantile; SD, standard deviation of the mean.

### Descriptive findings of the Fugl-Meyer Motor assessment

Between June and September 2021, 50 FMMA ratings were carried out by both assessors. The difference in median total scores between assessors was less than three points for the FMMA-UE and one point for the FMMA-LE. The median values with the corresponding first and third quantiles of the two assessors’ total FMMA scores are shown in Table 2.

**Table 2:** FMMA median and quantile values of the two assessors.

FMMA (*n* = 50)	Rater 1	Rater 2
FMMA-UE total score, median (Q1-Q3)	40.00 (27.75–51.00)	42.50 (25.50–54.75)
FMMA-LE total score, median (Q1-Q3)	24.00 (21.00–28.00)	23.00 (19.25–25.75)

FMMA, Fugl-Meyer Motor assessment; FMMA-LE, Fugl-Meyer Motor assessment lower extremity; FMMA-UE, Fugl-Meyer Motor assessment upper extremity; n, number of subjects; Q1-Q3, Quantile 1 to Quantile 3.

### Primary endpoint

All interrater reliability parameters at the overall score and subscale levels of the FMMA are shown in Table 3.

**Table 3:** Interrater reliability parameters of the German version of the FMMA at the overall score level.

Score / Subscale	ICC (95% CI)	Weighted Cohen’s kappa (95% CI)	Spearman’s rho (95% CI)
Total score FMMA	**0.98 (0.96–0.99)**		0.93 (0.85–0.97)
Total score FMMA-UE	**0.98 (0.96–0.99)**		0.93 (0.85–0.97)
Total score FMMA-LE	**0.85 (0.70–0.92)**		0.75 (0.60–0.87)
UE subscale: proximal part	0.96 (0.91–0.98)		0.88 (0.76–0.95)
UE subscale: wrist	0.95 (0.82–0.98)	0.88 (0.88–0.88)	0.94 (0.89–0.97)
UE subscale: hand	0.95 (0.91–0.97)	0.91 (0.91–0.91)	0.91 (0.79–0.97)
UE subscale: coordination	0.94 (0.61–0.98)	0.89 (0.84–0.94)	0.91 (0.83–0.96)
LE subscale: hip, knee, ankle	0.80 (0.64–0.88)		0.61 (0.37–0.79)
LE subscale: coordination	0.77 (0.12–0.91)	0.62 (0.42–0.83)	0.78 (0.60–0.89)

FMMA, Fugl-Meyer Motor assessment; ICC, intraclass correlation coefficient; LE, lower extremity; Spearman’s rho, Spearman’s rank-order correlation coefficient, in all cases with *p*-values < 0.0001; UE, upper extremity; 95% CI, 95% confidence interval.

For all total scores (FMMA-UE, FMMA-LE and entire FMMA) as well as for the proximal part subscale of the FMMA-UE and the hip, knee, ankle subscale of the FMMA-LE, the ICC values were between 0.80 (95% CI 0.64–0.88) for volitional movement within flexor and extensor synergies of the lower extremity and 0.98 (95% CI 0.96–0.99) for the total scores of the FMMA-UE. The total scores of the entire FMMA were very similar at 0.98 (95% CI 0.96–0.99). Using Koo and Li′s (27) classification for the 95% CI of the ICC values, the reliability of the meaningful subscales as well as that of the total scores (values written bold in Table 3) was classified as moderate to excellent.

Weighted Cohen’s kappa values ranged from 0.62 (95% CI 0.42–0.83) for the coordination subscales of the FMMA-LE to 0.91 (95% CI 0.91–0.91) for the hand subscales of the FMMA-UE. Using Landis & Koch (1979) (31) benchmarking for kappa statistics, the strength of agreement was found to be moderate to almost perfect.

The Spearman’s rank-order correlation coefficients for the total score and subscale levels ranged from 0.61 to 0.94 (median 0.91), with the lowest value for the hip, knee, ankle subscales of the FMMA-LE (values <0.7). The highest values were obtained for the FMMA-UE total scores, the total scores of the entire FMMA, and the wrist, hand, and the coordination subscales of the FMMA-UE (values >0.9). All *p*-values of Spearman’s rank-order correlation coefficients were smaller than 0.001.

### Secondary endpoints

**Figure 4:**
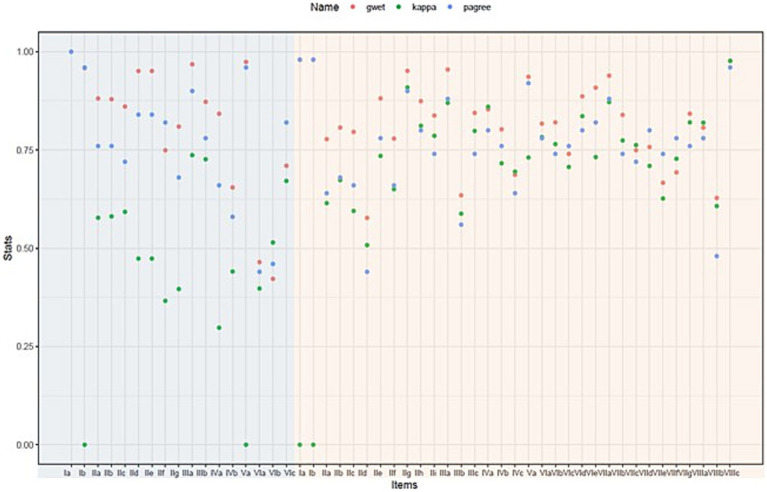
Graphical representation comparing percentage agreement, weighted Cohen’s kappa, and Gwet’s AC2 for each item. The items on light blue background are part of the FMMA-LE, those on light red of the FMMA-UE. Individual measurement points overlap for item Ib of the FMMA-LE and items Ia, Ib, VIa, and VIIIc of the FMMA-UE. FMMA-LE, Fugl-Meyer Motor assessment lower extremity; FMMA-UE, Fugl-Meyer Motor assessment upper extremity; gwet, Gwet’s AC1/AC2 coefficient; kappa, weighted Cohen’s kappa coefficient; pagree, percentage agreement.

All item-based interrater reliability parameters of the German version of the FMMA-UE are summarized in Table 4 and those of the FMMA-LE in Table 5. A graphical representation comparing all three item-level measures of the FMMA is shown in Figure 4.

**Table 4:** Item-based interrater reliability parameters of the German version of the FMMA-UE.

Item	Percentage agreement	Weighted Cohen’s kappa (95% CI)	Gwet’s AC1/AC2 (95% CI)
Ia. Biceps reflex	98	0.00 (0.00–0.00)	0.98 (0.94–1.00)
Ib. Triceps reflex	98	0.00 (0.00–0.00)	0.98 (0.94–1.00)
IIa. Shoulder: retraction	64	0.61 (0.42–0.81)	0.78 (0.69–0.87)
IIb. Shoulder: elevation	68	0.67 (0.48–0.86)	0.81 (0.72–0.90)
IIc. Shoulder: abduction	66	0.59 (0.39–0.80)	0.80 (0.71–0.88)
IId. Shoulder: external rotation	44	0.51 (0.27–0.75)	0.58 (0.44–0.72)
IIe. Elbow: flexion	78	0.73 (0.73–0.73)	0.88 (0.81–0.95)
IIf. Forearm: supination	66	0.65 (0.50–0.80)	0.78 (0.69–0.87)
IIg. Shoulder: adduction/IR	90	0.91 (0.91–0.91)	0.95 (0.91–1.00)
IIh. Elbow: extension	80	0.81 (0.75–0.88)	0.87 (0.8–0.95)
IIi. Forearm: pronation	74	0.79 (0.74–0.83)	0.84 (0.75–0.93)
IIIa. Hand to lumbar spine	88	0.87 (0.87–0.87)	0.95 (0.91–1.00)
IIIb. Shoulder: flexion 0–90°	56	0.59 (0.36–0.82)	0.63 (0.49–0.78)
IIIc. Pronation/supination 90°	74	0.80 (0.71–0.89)	0.84 (0.76–0.93)
IVa. Shoulder: abduction 0–90°	80	0.86 (0.79–0.93)	0.85 (0.77–0.94)
IVb. Shoulder: flexion 90–180°	76	0.72 (0.57–0.87)	0.80 (0.67–0.94)
IVc. Pronation/supination 0°	64	0.69 (0.51–0.88)	0.69 (0.54–0.84)
Va. Biceps/triceps reflexes	92	0.73 (0.73–0.73)	0.94 (0.86–1.00)
VIa. Wrist: dorsiflexion 90°	78	0.78 (0.63–0.94)	0.82 (0.69–0.95)
VIb. Wrist: dorsi−/volar flexion 90°	74	0.77 (0.61–0.92)	0.82 (0.74–0.90)
VIc. Wrist: dorsiflexion 0°	76	0.71 (0.58–0.83)	0.74 (0.55–0.93)
VId. Wrist: dorsi−/volar flexion 0°	80	0.84 (0.84–0.84)	0.89 (0.80–0.97)
VIe. Wrist: circumduction 90°	82	0.73 (0.73–0.73)	0.91 (0.85–0.97)
VIIa. Mass flexion	88	0.87 (0.87–0.87)	0.94 (0.89–0.99)
VIIb. Mass extension	74	0.77 (0.67–0.88)	0.84 (0.76–0.92)
VIIc. Hook grasp	72	0.76 (0.71–0.82)	0.75 (0.58–0.92)
VIId. Thumb adduction	80	0.71 (0.54–0.88)	0.76 (0.57–0.95)
VIIe. Pincer grasp	74	0.63 (0.45–0.80)	0.67 (0.44–0.89)
VIIf. Cylinder grasp	78	0.73 (0.59–0.87)	0.69 (0.48–0.91)
VIIg. Spherical grasp	76	0.82 (0.78–0.86)	0.84 (0.75–0.94)
VIIIa. Tremor upper extremity	78	0.82 (0.73–0.90)	0.81 (0.67–0.95)
VIIIb. Dysmetria upper extremity	48	0.61 (0.48–0.74)	0.63 (0.51–0.75)
VIIIc. Speed upper extremity	96	0.98 (0.98–0.98)	0.98 (0.94–1.00)

FMMA-UE, Fugl-Meyer Motor assessment upper extremity; IR, internal rotation; 95% CI, 95% confidence interval.

**Table 5:** Item-based interrater reliability parameters of the German version of the FMMA-LE.

Item	Percentage agreement	Weighted Cohen’s kappa (95% CI)	Gwet’s AC1/AC2 (95% CI)
Ia. Patellar reflex	100	-	-
Ib. Achilles reflex	96	0.00 (0.00–0.00)	0.96 (0.90–1.00)
IIa. Hip: flexion	76	0.58 (0.55–0.60)	0.88 (0.82–0.95)
IIb. Knee: flexion	76	0.58 (0.58–0.58)	0.88 (0.82–0.95)
IIc. Ankle: dorsiflexion	72	0.59 (0.59–0.59)	0.86 (0.78–0.94)
IId. Hip: extension	84	0.47 (−0.71–1.00)	0.95 (0.91–0.99)
IIe. Hip: adduction	84	0.47 (−0.71–1.00)	0.95 (0.91–0.99)
IIf. Knee: extension	82	0.37 (0.04–0.69)	0.75 (0.57–0.93)
IIg. Ankle: plantar flexion	68	0.40 (−0.13–0.93)	0.81 (0.68–0.94)
IIIa. Knee flexion sitting	90	0.74 (0.74–0.74)	0.97 (0.94–1.00)
IIIb. Ankle dorsiflexion sitting	78	0.73 (0.73–0.73)	0.87 (0.80–0.94)
IVa. Knee flexion standing	66	0.30 (−0.40–1.00)	0.84 (075–0.94)
IVb. Ankle dorsiflexion standing	58	0.44 (0.17–0.71)	0.65 (0.46–0.85)
Va. Patellar/achilles reflexes	96	0.00 (0.00–0.00)	0.97 (0.93–1.00)
VIa. Dysmetria lower extremity	44	0.40 (0.22–0.58)	0.46 (0.24–0.69)
VIb. Tremor lower extremity	46	0.51 (0.35–0.68)	0.42 (0.19–0.65)
VIc. Speed lower extremity	82	0.67 (0.48–0.86)	0.71 (0.49–0.93)

FMMA-LE, Fugl-Meyer Motor assessment lower extremity; 95% CI, 95% confidence interval.

Most of the assessor agreements were above 0.75 for most of the items. The three different statistical parameters (percentage agreement, weighted Cohen’s kappa coefficients, Gwet’s AC1/AC2 coefficients) shown in the graph were far apart for some items, indicating a high heterogeneity, while they were close for others. It can also be seen that the parameter distances were generally larger for the lower extremity items than for the upper extremity items.

Percentage agreement values for upper extremity items ranged from 44 to 98% (median 77%), with the highest data for reflex activities (values >95%) and the lowest for one component each of the flexor synergy and the coordination subscale (values <50%). Agreement values for the FMMA-LE were generally similar to those of the FMMA-UE and ranged from 44 to 100%. The highest agreement was found for the presence or absence as well as the quality of reflex activity (values >95%) and the lowest for two components of the coordination subscale (values <50%).

As presented in Table 4, weighted Cohen’s kappa coefficients of the upper extremity items ranged from 0.00 (95% CI 0.00–0.00) to 0.98 (95% CI 0.98–0.98) with a median of 0.73. Based on the benchmarking of Landis & Koch (1977) (31), the strength of the agreement could be classified as slight to almost perfect. For the FMMA-LE (see Table 5), the lowest kappa values were 0.00 (95% CI 0.00–0.00) for item Ib. as well as for item Va. and the highest was 0.74 (95% CI 0.74–0.74) for one component of the tasks performed in a sitting position. The median was 0.47. Thus, the degree of agreement was slight to substantial.

In most cases, the Gwet’s AC1/AC2 coefficients were higher than the weighted Cohen’s kappa coefficients. Based on these Gwet’s AC1/AC2 values (median for the items of the entire FMMA 0.84, range 0.42–0.98; median for the items of the FMMA-UE 0.82, range 0.58–0.98; median for the items of the FMMA-LE 0.87, range 0.42–0.97), moderate to almost perfect agreement according to the classification of Landis and Koch (1977) (31) was found for the FMMA-UE, while it was also moderate to almost perfect for the lower extremity.

## Discussion

The results indicate the following key findings: (i) The total scores of the entire FMMA show excellent interrater reliability of the German FMMA version across different health professions. This makes it suitable for quantitative measurement of stroke recovery in both clinical practice and research. (ii) Interrater reliability at the item level was lower than in comparable studies with FMMA versions in other languages, leaving room for potential improvement in this area.

### Interrater reliability at the overall score level

For the total scores of the entire FMMA, which includes both the FMMA-UE and FMMA-LE, the ICC was 0.98 (95% CI 0.96–0.99), which is considered excellent (27). This finding is consistent with studies that investigated the interrater reliability of the English version of the FMMA in different settings (12, 13, 26).

### Item-level interrater reliability

The percentage agreement values in the present study, which ranged from 44 to 98% for the FMMA-UE and from 44 to 100% for the FMMA-LE, were lower than those of the Colombian Spanish version of Hernández et al. (2019, 2020) (24, 25), which ranged from 88 to 100% for the FMMA-UE and FMMA-LE. The level of agreement for the items of the FMMA-UE and FMMA-LE in the transculutural/cross-cultural translation and validation studies was above 70% for an Italian version (23) and for a Danish version (22). Both working groups classified an agreement of ≥70% as satisfactory (22, 23). In contrast, the agreement values for our German version were below 70% for eight items of the FMMA-UE (seven of them within the proximal part subscale) and five items of the FMMA-LE (two of them within the coordination subscale).

Particularly noticeable are the lower percentage agreement values of the respective three items from the coordination subscales of the FMMA-UE and FMMA-LE compared to the data of the above-mentioned articles. In this study, the FMMA-UE coordination item values ranged from 48 to 96% (with the lowest value for dysmetria followed by tremor) and FMMA-LE values ranged from 44 to 82%, whereas the reference studies reported FMMA-UE coordination item values of at least 80% and FMMA-LE values of at least 70% (22–25). These discrepancies raise the question of whether the items of the coordination subscale of the German FMMA should be defined more specifically.

Another explanation for the lower interrater reliability values at the item level in the present project could be that the assessors of the reference studies were therapists (22–25). In the present project the assessors consisted of a physician and a physiotherapist. At the item level, profession-specific differences in rating may well be apparent.

### Implications for research and clinical practice

According to expert recommendations (5, 6, 8), the FMMA should be implemented as important assessment for the body function and structure domain of the International Classification of Functioning, Disability and Health (ICF) throughout the continuum of stroke care to optimize the quality of the rehabilitation pathway. The results of the present project make a small but important contribution on this way.

To ensure a consistent and uniform application of the FMMA, a clear, standardized training and refresher training structure as well as a lively exchange between assessors during the training process are of great importance. These elements are largely similar to the procedures used in our training setting. Therefore, and in line with See et al. (26), we recommend the creation of instructional videos as well as test patient videos to compare scoring as a supplement to FMMA presence training in small groups with an expert and standardized assessment forms.

Based on the proposed measures, the assessment forms of the German version of the FMMA can be further developed and the training structure can be adapted. In the future, international standardization and harmonization of FMMA protocols might be useful.

### Strengths

To the best of our knowledge, this is the first reliability study with a cross-sectional design at a predefined measurement time point in which two assessors evaluated the interrater reliability of a German FMMA version using video recordings of 50 individuals after stroke. Except for the studies by Hernández et al. (24, 25) with 60 stroke patients, all selected reference studies with similar populations had smaller sample sizes (12, 13, 21–23, 26, 32). Furthermore, the consistency of the ICC values across different calculation methods indicated the robustness of our key findings.

The assessors of the current project belong to two different health professions (a physician and a physical therapist), which can be seen as a strength considering that the FMMA is meant to be used more widely in the future. Therefore, and for the envisioned higher acceptance of the FMMA as key motor recovery assessment tool, a high reliability across different professions is essential. Another strength was that the video recordings could be evaluated remotely, avoiding repetitions of the FMMA, which would have introduced the risk of bias due to learning effects. Furthermore, the video approach may allow centralized adjudication within the multicentre ESTREL trial and could improve the quality of future stroke recovery and rehabilitation studies.

### Limitations

We are aware of the following limitations of our project. Firstly, the generalisability of the findings based on video recordings with only two assessors has not been demonstrated. A future statistical reliability analysis should incorporate the original FMMA scores from the ESTREL database obtained from real measurements at the time of videotaping in the presence of patients. In this way, the original on-site FMMA ratings might be compared with the ratings of the two assessors based on the videotaped FMMA. This would allow additional comparison of interrater reliability with that reported in the literature based on FMMA ratings in the presence of patients.

Secondly, different statistical approaches to calculating interrater reliability were described in the literature. The parallel calculation of Gwet’s AC1/AC2 coefficients for the item level of the FMMA can be considered a useful complement to the weighted Cohen-Kappa coefficients. The statistic of Gwet, in turn, is not well known because studies of interrater reliability in the current research field rarely report these coefficients. The comparability of study results is important in this context, which is why the use of Svensson’s method (33), for example, should be considered in future cross-cultural translations and adaptations. Likewise, the determination of systematic disagreement would be an interesting approach. For example, the tasks actively performed by patients with combined movement levels and directions, which are difficult to assess from only one perspective, tended to reflect more systematic inconsistencies. This was evident in some large movement tasks as well as in the evaluation of dysmetria of the FMMA-UE, but also in the standing items and coordination tasks (dysmetria and tremor) of the FMMA-LE. Thirdly, the results of our study are not necessarily applicable to populations other than stroke patients and to assessors from other health professions (e.g., study nurses).

## Conclusion

The FMMA appears to be a highly reliable measuring instrument at the overall score level for assessors from different health professions. The FMMA total scores seem to be suitable for the quantitative measurement of stroke recovery in both clinical practice and research, although there is potential for improvement at the item level.

## Data availability statement

The datasets generated for this study are available on request to the corresponding author.

## Ethics statement

All participants had provided their informed consent to participate in ESTREL, in which videotaping of the FMMA is mentioned. Ethical review and approval were not required for the study on human participants in accordance with the local legislation and institutional requirements (BASEC-Nr. Req.-2020–00443). Written informed consent was obtained from the individuals for the publication of any potentially identifiable images or data included in this article.

## Author contributions

KW: Writing – review & editing, Writing – original draft. AS: Writing – review & editing. LM: Writing – review & editing. JK: Writing – review & editing. CT: Writing – review & editing. AL: Writing – review & editing. JH: Writing – review & editing, Writing – original draft. SE: Writing – review & editing, Writing – original draft.
