# Supplementary figures and images for: Interrater reliability of the Fugl-Meyer Motor assessment in stroke patients: a quality management project within the ESTREL study
Source: Front Neurol. 2024 Apr 8;15:1335375. doi: 10.3389/fneur.2024.1335375 (PMC11034517; doi:10.3389/fneur.2024.1335375)

**Table S1:**


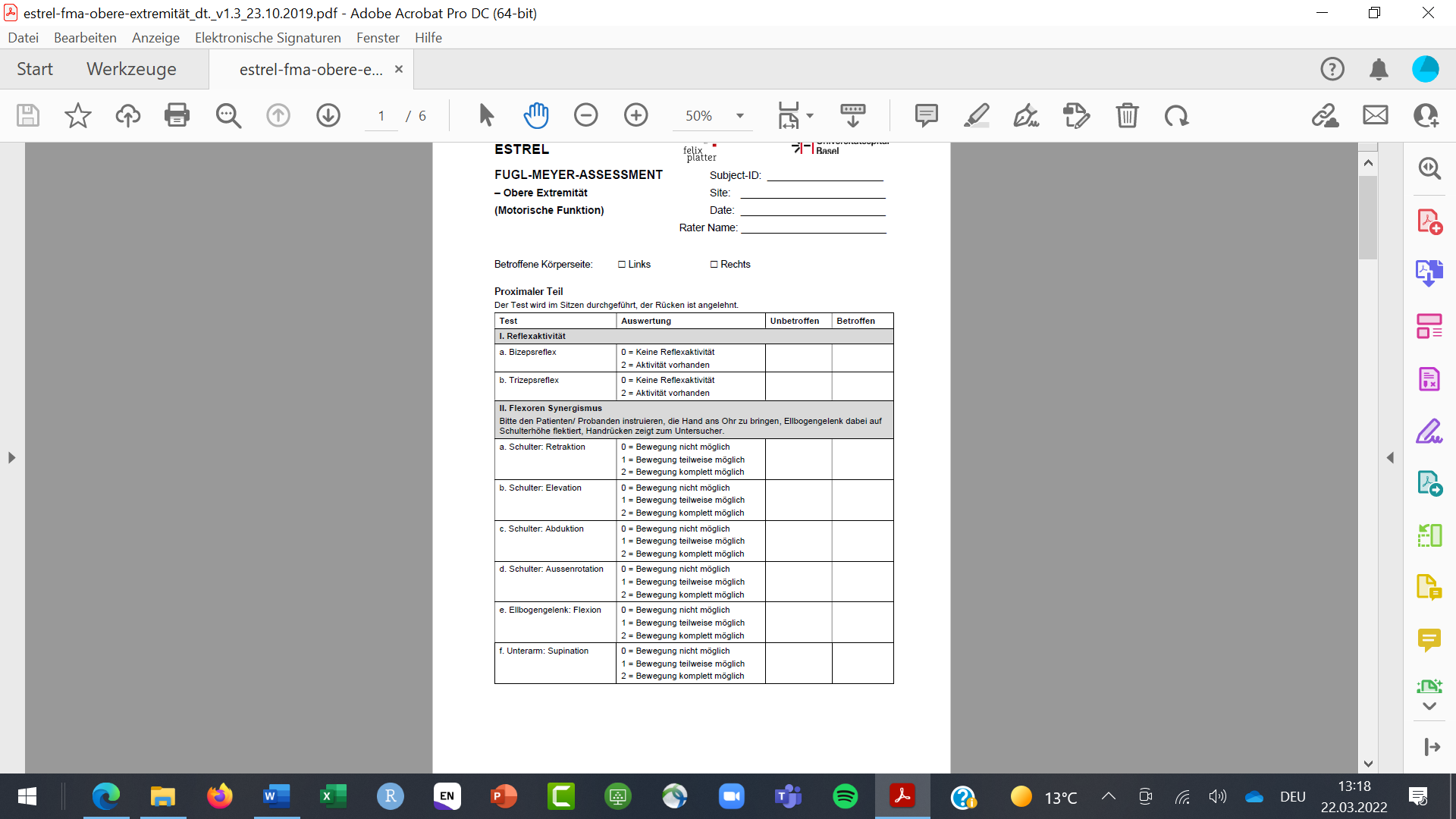


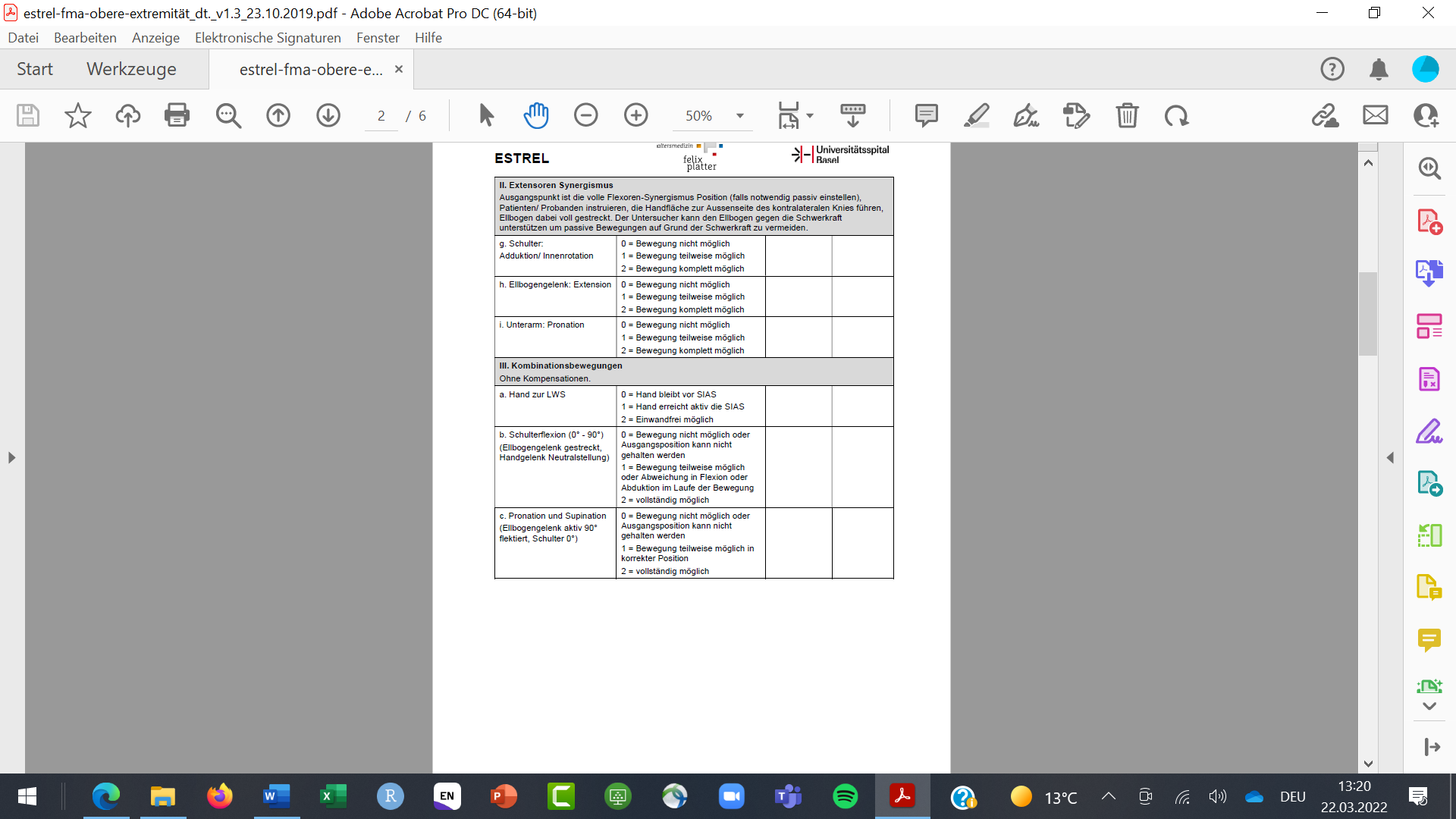


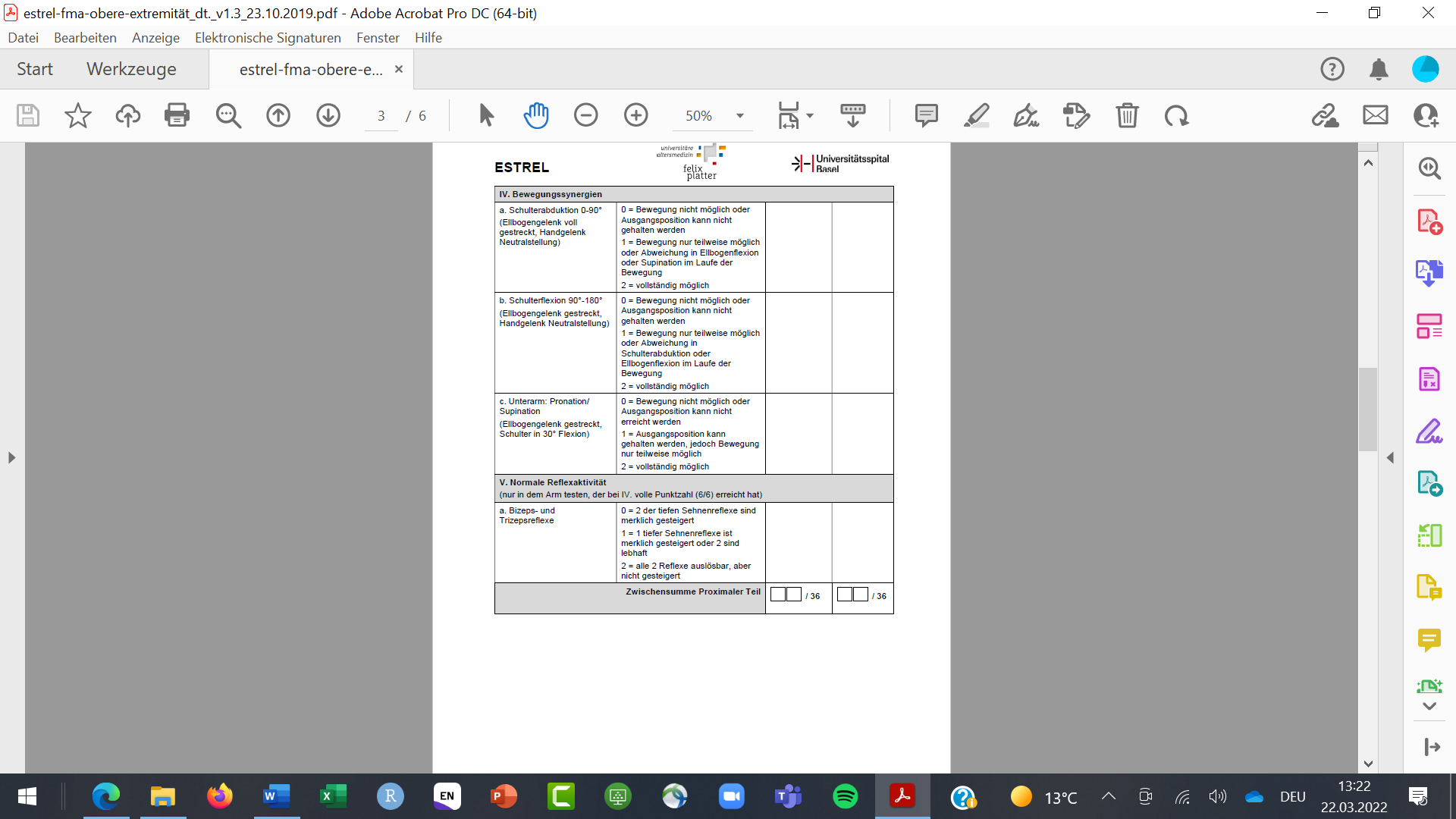


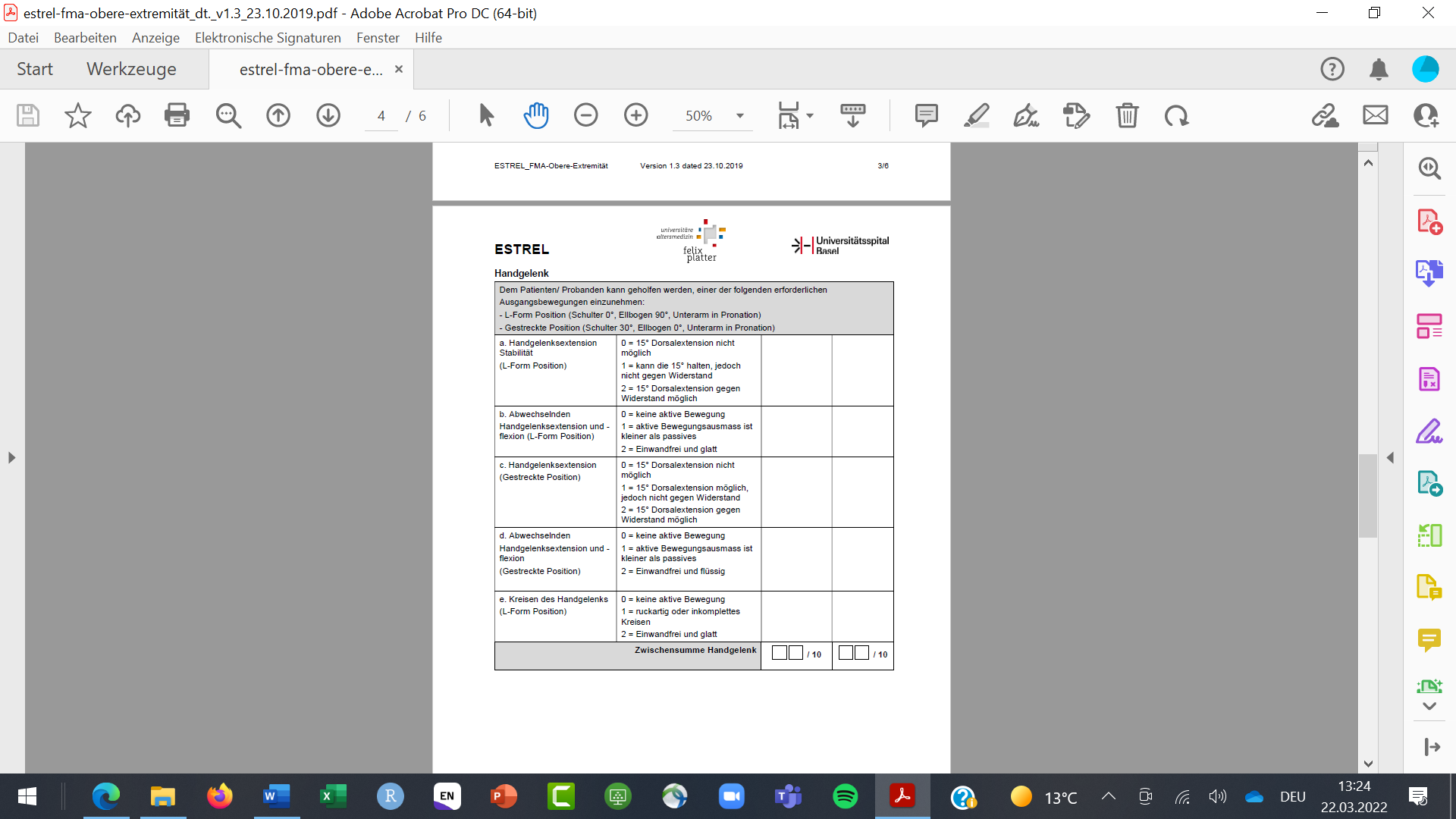


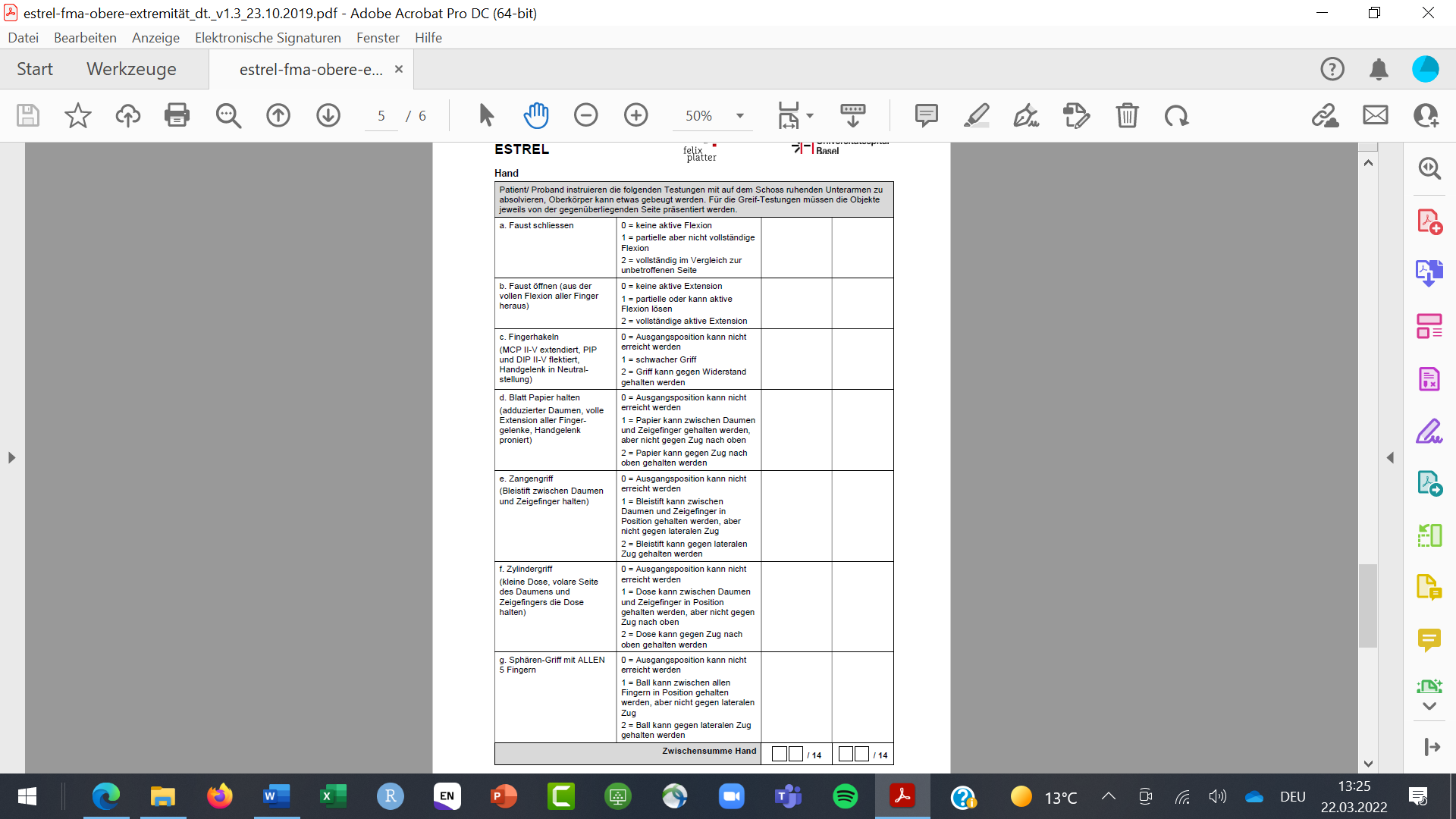


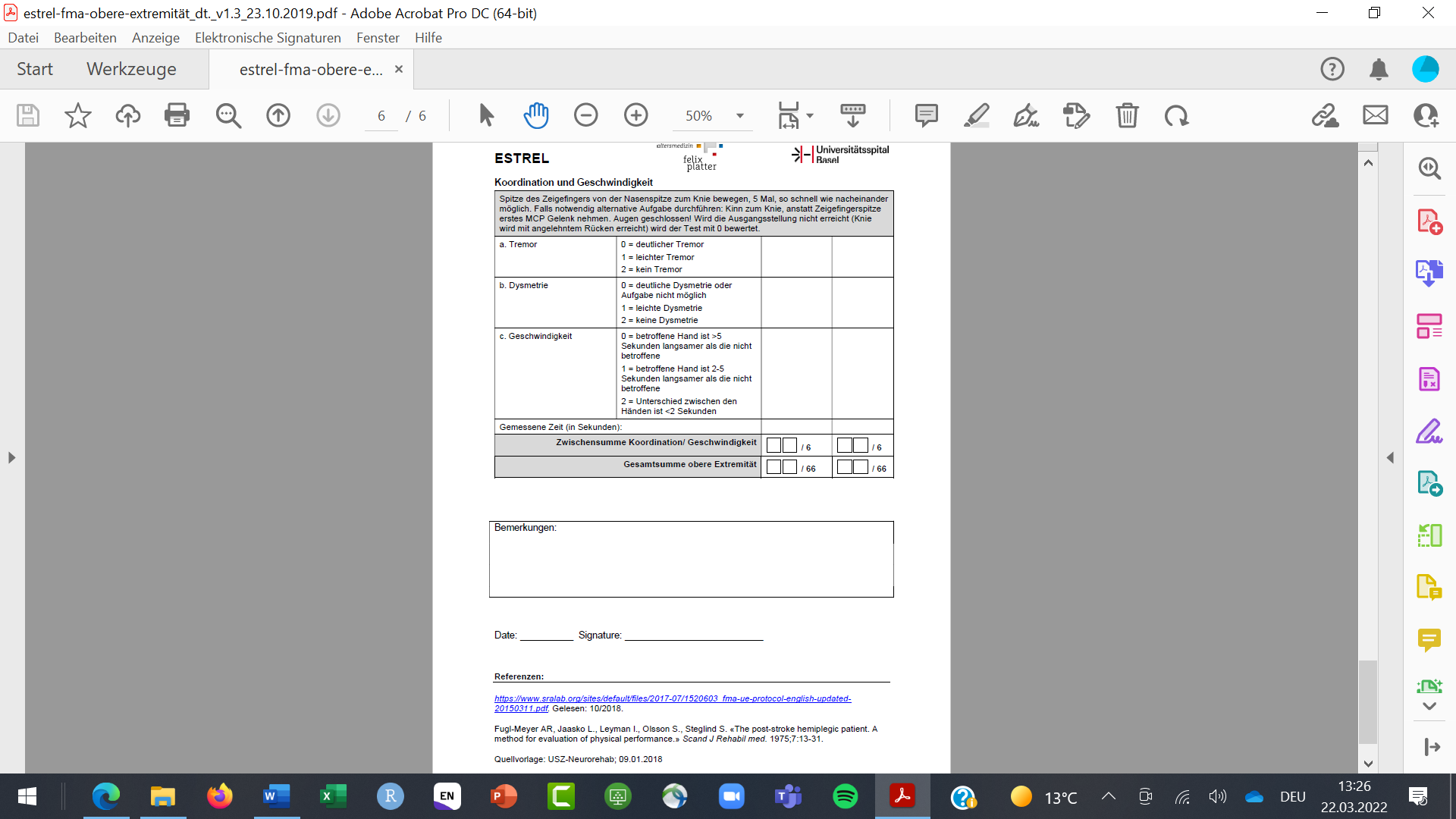


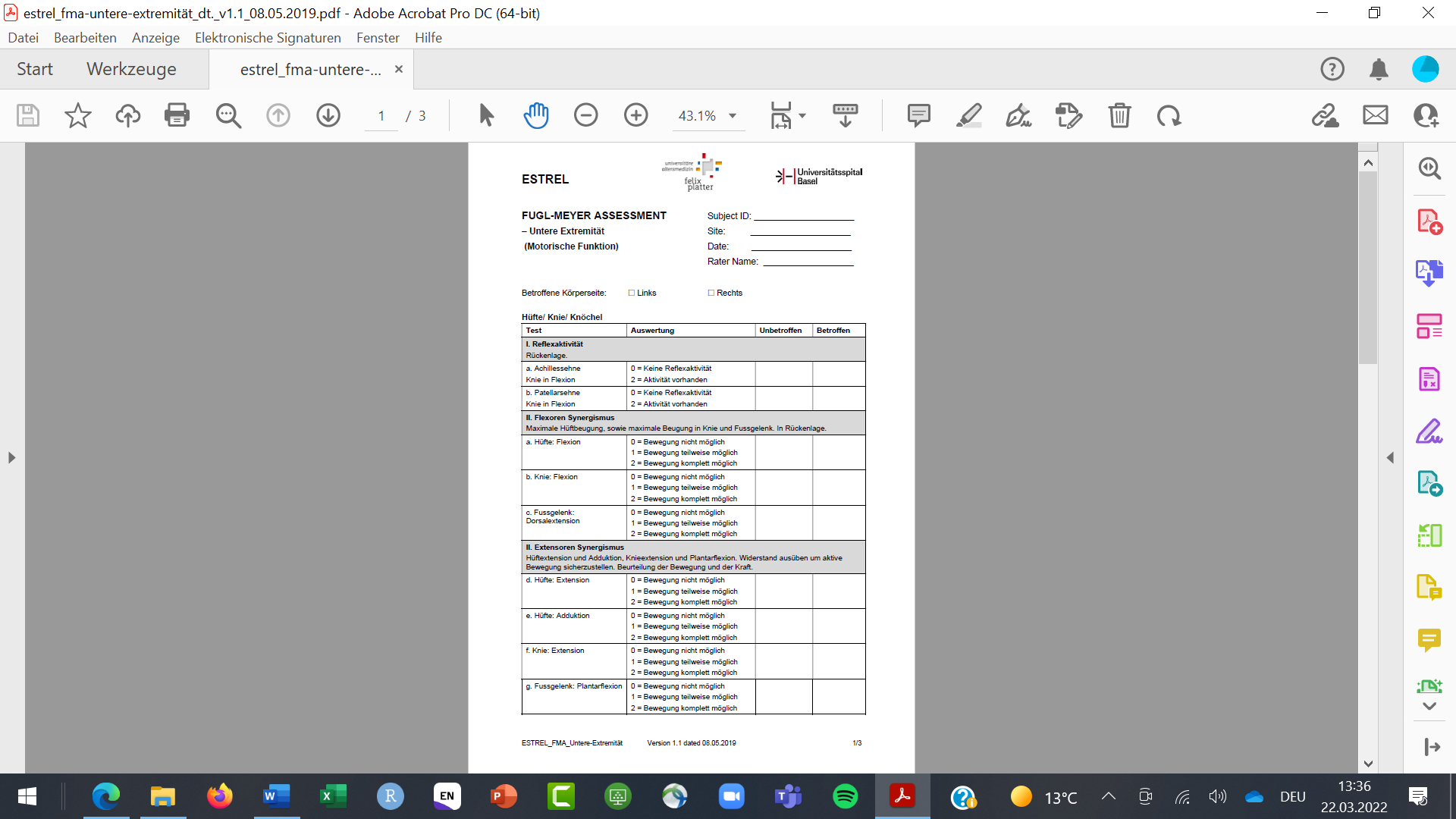


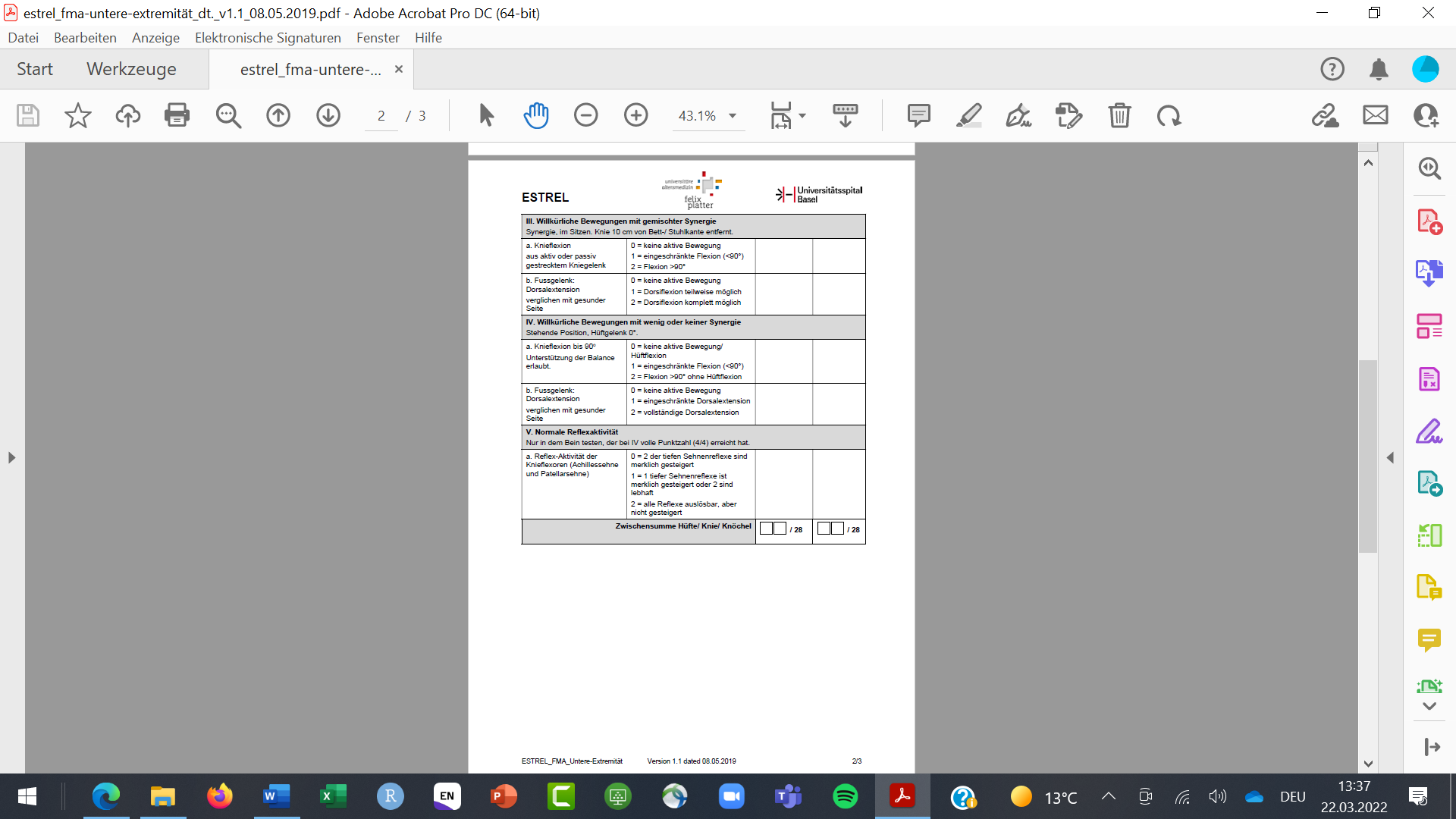


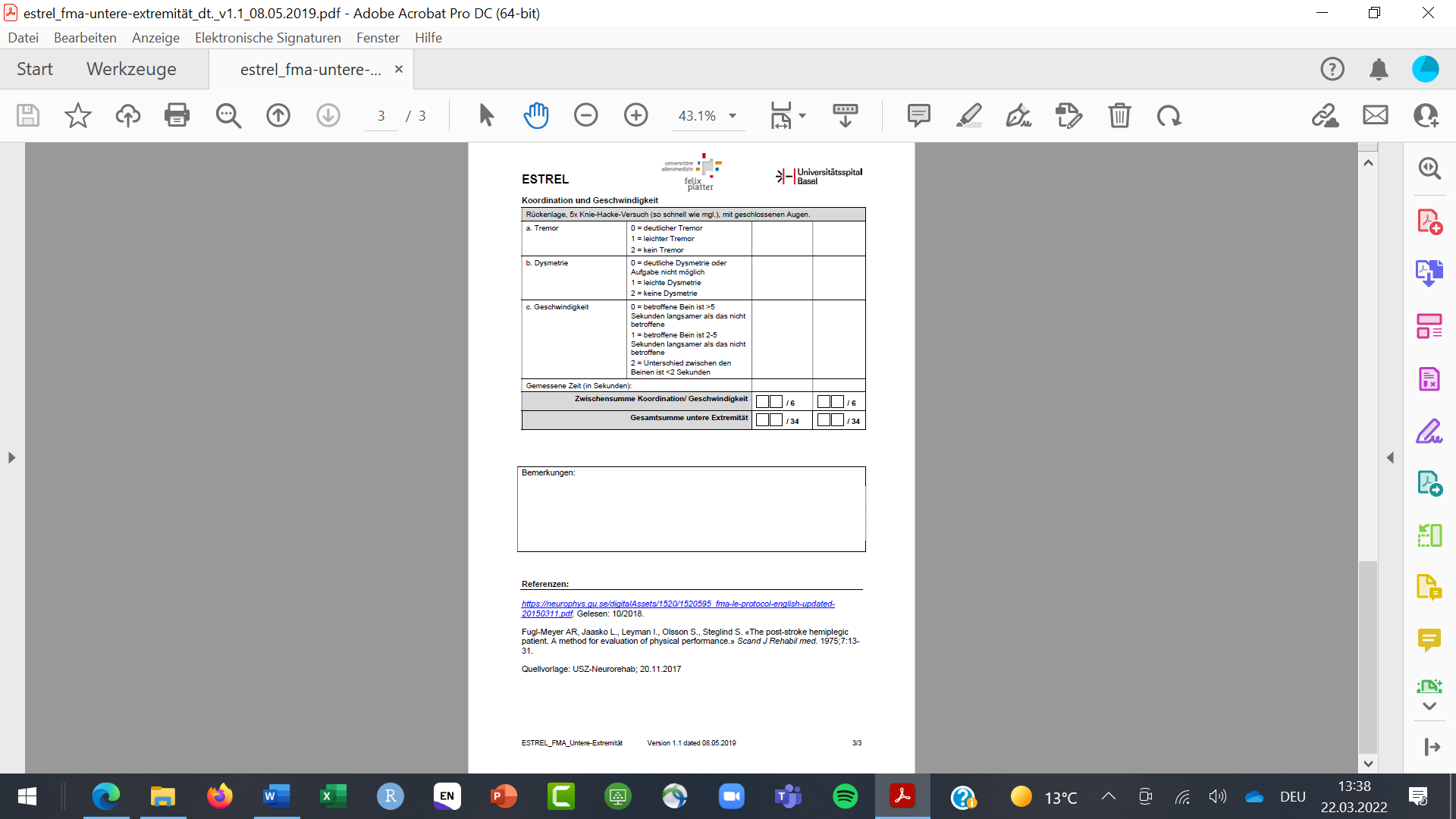

Supplement: Supplementary file 1 [file Table_1.DOCX]
