# Supplementary material for: Interrater reliability of the Fugl-Meyer Motor assessment in stroke patients: a quality management project within the ESTREL study
Source: Front Neurol. 2024 Apr 8;15:1335375. doi: 10.3389/fneur.2024.1335375 (PMC11034517; doi:10.3389/fneur.2024.1335375)
Supplement: Supplementary file 2 [file Table_2.DOCX]

**TABLE S2 |** Classification of ICC estimates, respectively their 95% CI. Adapted from Koo & Li, 2016, p. 161 (27).

| ICC (95% CI) | Reliability |
| --- | --- |
| < 0.5 | Poor |
| 0.5-0.75 | Moderate |
| 0.75-0.9 | Good |
| > 0.9 | Excellent |
