# Supplementary material for: Interrater reliability of the Fugl-Meyer Motor assessment in stroke patients: a quality management project within the ESTREL study
Source: Front Neurol. 2024 Apr 8;15:1335375. doi: 10.3389/fneur.2024.1335375 (PMC11034517; doi:10.3389/fneur.2024.1335375)
Supplement: Supplementary file 3 [file Table_3.DOCX]

**TABLE S3 |** Classification for kappa statistics. Adapted from Landis & Koch, 1977, p. 165 (31).

| Kappa statistic | Strength of agreement |
| --- | --- |
| < 0.00 | Poor |
| 0.00-0.20 | Slight |
| 0.21-0.40 | Fair |
| 0.41-0.60 | Moderate |
| 0.61-0.80 | Substantial |
| 0.81-1.00 | Almost perfect |
